# Adapting hospital capacity to meet changing demands during the COVID-19 pandemic

**DOI:** 10.1186/s12916-020-01781-w

**Published:** 2020-10-16

**Authors:** Ruth McCabe, Nora Schmit, Paula Christen, Josh C. D’Aeth, Alessandra Løchen, Dheeya Rizmie, Shevanthi Nayagam, Marisa Miraldo, Paul Aylin, Alex Bottle, Pablo N. Perez-Guzman, Azra C. Ghani, Neil M. Ferguson, Peter J. White, Katharina Hauck

**Affiliations:** 1 MRC Centre for Global Infectious Disease Analysis and Abdul Latif Jameel Institute for Disease and Emergency Analytics, Imperial College London, Norfolk Place, London, W2 1PG UK; 2 Centre for Health Economics & Policy Innovation, Department of Economics & Public Policy, Imperial College Business School, Imperial College London, London, UK; 3 Dr Foster Unit, Department of Primary Care and Public Health, Imperial College London, London, UK; 4 NIHR Health Protection Research Unit in Healthcare Associated Infections and Antimicrobial Resistance, Imperial College London, London, UK; 5 NIHR Health Protection Research Unit in Modelling and Health Economics, Imperial College London, London, UK; 6 Modelling and Economics Unit, National Infection Service, Public Health England, London, UK

**Keywords:** Hospital capacity, Interventions, General & acute, Critical care, Elective surgery, COVID-19

## Abstract

**Background:**

To calculate hospital surge capacity, achieved via hospital provision interventions implemented for the emergency treatment of coronavirus disease 2019 (COVID-19) and other patients through March to May 2020; to evaluate the conditions for admitting patients for elective surgery under varying admission levels of COVID-19 patients.

**Methods:**

We analysed National Health Service (NHS) datasets and literature reviews to estimate hospital care capacity before the pandemic (pre-pandemic baseline) and to quantify the impact of interventions (cancellation of elective surgery, field hospitals, use of private hospitals, deployment of former medical staff and deployment of newly qualified medical staff) for treatment of adult COVID-19 patients, focusing on general and acute (G&A) and critical care (CC) beds, staff and ventilators.

**Results:**

NHS England would not have had sufficient capacity to treat all COVID-19 and other patients in March and April 2020 without the hospital provision interventions, which alleviated significant shortfalls in CC nurses, CC and G&A beds and CC junior doctors. All elective surgery can be conducted at normal pre-pandemic levels provided the other interventions are sustained, but only if the daily number of COVID-19 patients occupying CC beds is not greater than 1550 in the whole of England. If the other interventions are not maintained, then elective surgery can only be conducted if the number of COVID-19 patients occupying CC beds is not greater than 320. However, there is greater national capacity to treat G&A patients: without interventions, it takes almost 10,000 G&A COVID-19 patients before any G&A elective patients would be unable to be accommodated.

**Conclusions:**

Unless COVID-19 hospitalisations drop to low levels, there is a continued need to enhance critical care capacity in England with field hospitals, use of private hospitals or deployment of former and newly qualified medical staff to allow some or all elective surgery to take place.

## Background

The coronavirus disease (COVID-19) pandemic has placed severe strain on health systems worldwide, with large and rapid changes in demand for inpatient care. Caring for COVID-19 patients whilst maintaining treatment for patients with other conditions is a complex planning challenge. Ensuring safe and timely care to both COVID-19 patients and those with other conditions is a crucial aspect of England’s response to this crisis [1].

In England, a range of interventions has been implemented to increase hospital capacity in response to the pandemic. Implemented hospital provision interventions included the procurement of equipment, the establishment of additional hospital facilities and the redeployment of staff and other resources. One of the most impactful interventions for freeing up bed capacity was the cancellation of elective surgery in March 2020 [2], which led to a backlog of patients requiring care. This is creating pressure on health services to conduct elective surgery, which needs to be addressed urgently [3]. Over March and April 2020, population-level measures to reduce transmission of SARS-CoV-2 have led to a gradual decline in the demand for hospital care by COVID-19 patients from a peak on 12 April, when 18,800 beds were occupied [4]. The challenge for healthcare planners now is planning capacity to treat non-COVID-19 conditions whilst maintaining the ability to respond to any potential future increases in demand for COVID-19 care.

Various tools have been developed to make projections of demand for care [5–8], but they do not assess the extent to which interventions suffice to address population care needs. Such guidance is crucial if elective surgery and other urgent care are to be re-introduced at pre-pandemic levels. The objectives of this study are threefold: first, to estimate available hospital capacity for emergency treatment of COVID-19 and other patients during the *surge* phase of the epidemic in England (March and April 2020); second, to evaluate the increase in capacity achieved via five hospital provision interventions (cancellation of elective surgery, set-up of field hospitals, use of private hospitals, deployment of former healthcare staff and deployment of newly qualified and final year nursing and medical students) during the *surge* phase; and third, to determine how to conduct elective surgery at pre-pandemic levels considering continued demand from COVID-19 patients during the *post-surge* phase.

## Methods

**Fig. 1:**
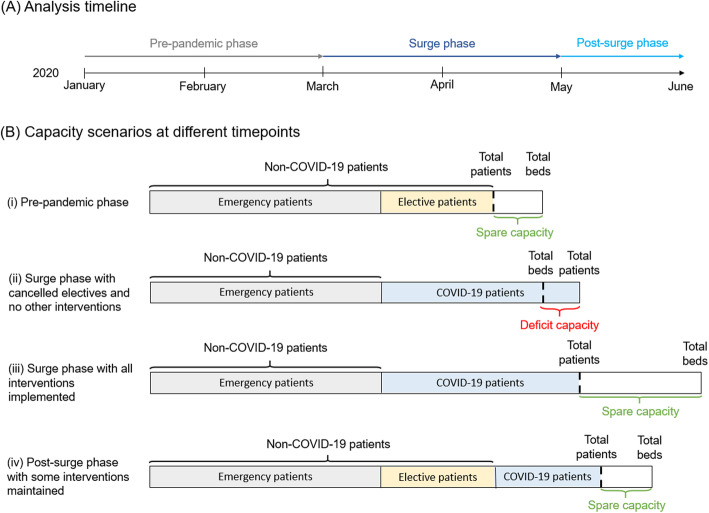
Schematic diagram of hospital capacity under different scenarios. **a** Timeline of the phases considered in the analysis. **b** Schematic illustration of bed capacity and occupancy partitioned non-COVID-19 and COVID-19 patients, and how this leads to either spare or deficit capacity, depending on the total number of beds available in the different phases and intervention scenarios. This is not drawn to scale. (i) Pre-pandemic phase, during which *baseline bed capacity* is defined as total beds, and baseline patient occupancy is defined as the number of these beds occupied, in the absence of hospital provision interventions and COVID-19 patients. (i) In the surge phase (ii and iii), all elective surgery was assumed to be cancelled, freeing up beds for COVID-19 patients. However, in (ii), this alone did not provide sufficient beds for all patients and thus there is deficit capacity. Other hospital provision interventions were used to increase the total number of beds in (iii) so that there was even spare capacity of beds. In the post-surge phase (iv), reductions in numbers of COVID-19 patients enables some elective surgery to resume, with the numbers of such patients who can be accommodated depending on the extent to which other interventions are maintained

We defined capacity in terms of staff, beds and ventilators (herein referred to as *resources*). Data inputs and sources can be found in Additional file 2 [4, 9–21]. The analysis considered changes to resources across three different time points: the pre-pandemic phase, the surge phase and the post-surge phase (Fig. 1a, Additional file 1).

The *pre-pandemic phase* considered capacity before the onset of the COVID-19 pandemic in England (pre-March 2020). During this phase, we assumed *baseline capacity*, which is estimated as the average number of resources, and baseline patient occupancy, which is the number of these baseline resources occupied, to be constant.

The *surge phase* referred to the period of March–April 2020, during which there was a large increase in the numbers of hospitalised COVID-19 cases, and interventions to increase hospital capacity were implemented. Throughout this second phase, we considered the impact of interventions on the spare capacity of resources, which is a function of the capacity and patient occupancy, to determine whether patients could access treatment. For this, we developed a model to estimate the corresponding number of COVID-19 patients that would have been able to be accommodated on top of expected non-COVID-19 demand in the pre-pandemic phase. To determine the threshold numbers of COVID-19 patients at which capacity requirements would be exceeded with implemented interventions, we used the model to evaluate the impact of these, both individually and in combination, on top of the baseline capacity and patient occupancy.

Finally, the *post-surge phase* began in May 2020. At this point, the number of hospitalised COVID-19 cases has been observed to gradually decline, and hospitals have considered how to safely provide care again for all patients requiring it, whilst also planning for possible future surges in COVID-19 case numbers. In this part of the analysis, we used the model to determine how the re-introduction of elective surgery could be enabled by changes to the hospital provision interventions.

Throughout, *spare capacity* was defined by the difference between the total resources available and the capacity to accommodate a given demand, as determined by patient occupancy numbers (Fig. 1b; Additional file 3). If negative, this reflects a deficit in capacity.

### Estimation of baseline capacity in pre-pandemic phase

The baseline capacity of overnight beds, nurses, junior doctors and senior doctors, split by general and acute (G&A) and critical care (CC), and ventilators, was estimated for England using National Health Service (NHS) data in the pre-pandemic phase [9–11, 13]. In England, hospital capacity and patient occupancy data are available by NHS trust level (Additional file 1). To account for seasonal fluctuations in capacity, adjusted with respect to seasonal fluctuations in expected demand, we assumed average daily numbers of beds and staff from April–June 2019. This period is most representative of what current capacity and occupancy would have been, without implementation of hospital provision interventions. CC bed numbers include beds in intensive care and high dependency units. We included G&A and CC beds and staff from all acute and community provider NHS trusts but excluded children’s trusts. CC paediatric beds and occupancy are distinguished from adult beds which was reflected in our estimates, but this distinction could not be made for G&A [9, 10]. However, the majority of hospitalised COVID-19 cases are adults and while some hospitals may have converted paediatric beds to treat adults, we do not anticipate this substantially altering the outcome of the analysis [22]. We further distinguished between senior and junior doctors to reflect the requirement of senior clinical decision-makers on wards. Staff numbers are considered in units of full-time equivalents (FTEs) to account for staff employed on a part-time basis or absent due to illness and the possibility of staff working in various wards. Electronic Staff Records (ESR) data were filtered for staff categories normally working on these wards. For example, midwives, general practitioners and paediatric staff were excluded. According to the number of beds in each trust, a weighted average of daily FTE was calculated for each staff category at a national level.

Staff-to-beds ratios specified by the Royal College of Nursing, the Royal College of Physicians and the Faculty of Intensive Care Medicine [16–18] were used to quantify required safe staffing levels per category. These were kept constant throughout the analysis. The baseline capacity of ventilators and other parameters in the model were derived from various sources (Additional file 2 [4, 9–21]).

### Capacity during the surge phase

#### COVID-19 variables

The observed peak number of hospitalised patients with confirmed COVID-19 recorded (as of 31 May 2020) was set as the maximum number of COVID-19 patients in this analysis [4, 23]. This occurred on 12 April 2020, when approximately 3100 and 15,700 COVID-19 patients were occupying CC and G&A beds, respectively (Additional file 2 [4, 9–21]). We estimated the absence rate of staff due to COVID-19 during this period from surveys of union members for nurses and doctors [19]. These rates were coupled with baseline absence rates, to calculate the number of available staff during the surge.

#### Hospital provision interventions

Interventions implemented in England during the surge phase were previously identified [24] through a review of NHS sources, the European Observatory’s Health System Response Monitor [25] as well as the public press and were included in the model if they could be quantified at a national level.

The expected impact of each intervention across all resources was calculated as percentage changes of the baseline based on an analysis of NHS England data [26, 27] and from various sources [28–30] (Additional file 3). The expected proportion of occupied beds freed up through cancellation of elective surgery was estimated from Hospital Episode Statistics (HES) data of the busiest month in hospitals in January 2019 [27]. This is considered a conservative estimate because this month is the busiest in terms of demand for care. Elective patients requiring hospital care on any average day pre-COVID-19 (herein referred to as elective patients) were defined as those classified as non-emergency, non-maternity and non-cancer in the dataset and considered only if admitted to hospital overnight. They were also stratified into CC and G&A.

#### Analysis

For the surge phase, the model was used to calculate the spare capacity of resources under varying numbers of adult COVID-19 and non-COVID-19 patients on a given day, accounting for COVID-19-related staff absence, staff-to-bed ratios and the proportion of CC patients requiring ventilation (Fig. 1; Additional file 2 [4, 9–21]; Additional file 3). The maximum number of COVID-19 patients that could be accommodated by each resource under different scenarios, namely, no interventions, each individual intervention and the combination of hospital provision interventions that was implemented (herein referred to as the *implemented intervention package*), was determined. This was compared with the estimated maximum number of COVID-19 patients at the observed peak number of hospitalised COVID-19 patients during the first pandemic wave in England. The limiting resources in national baseline capacity were identified as the resources accommodating the smallest number of COVID-19 patients in the absence of interventions. We further compared the magnitude of spare capacity or deficits in different resources under the different scenarios of interventions for the observed peak number of hospitalised COVID-19 patients.

### Reintroduction of elective patients in the post-surge phase

For the post-surge phase, we estimated the number of elective patients who could be accommodated under decreasing numbers of COVID-19 patients, for different intervention scenarios. This is referred to as *post-surge reintroduction of elective surgery patients*. This was facilitated by splitting non-COVID-19 patients into emergency patients, who continue to receive care throughout the pandemic, and elective patients (Fig. 1b). The number of patients that can be accommodated was determined by the number of patients for which all necessary resource categories displayed spare capacity (i.e. a non-negative value). Hospital provision interventions were assessed for their potential long-term feasibility based on official recommendations for the second phase of the NHS response to COVID-19 [4].

Both the number of COVID-19 patients and number of elective patients were varied, with the number of COVID-19 patients being reduced from the observed maximum in 10% intervals. This was done to consider scenarios of 0 to 100% of the maximum applied to both CC and G&A COVID-19 patients. We assumed that elective patients requiring G&A and CC will be introduced simultaneously. Using the previous analysis of HES and baseline occupancy data [9, 10, 27], we derived the expected number of elective patients that could be accommodated based on pre-pandemic demand and quantified a linear relationship between the number of elective patients in G&A and in CC (Additional file 3). Therefore, the daily number of G&A elective patients was varied in bands of 500, and the equivalent value for CC derived via this relationship.

All analysis was undertaken on R and is available publicly on Github.^1^

### Patient and public involvement

This research involved evaluating the impact of strategies already adopted by the NHS, and therefore, research questions, outcome measures and dissemination of study results were not developed or informed by patient or public involvement.

## Results

### Spare capacity in the pre-pandemic phase

We estimated that before the COVID-19 pandemic (pre-March 2020), there was a daily spare capacity of 817 CC beds, 9769 G&A beds, 6757 ventilators, 642 CC nurses, 14,394 G&A nurses, 745 CC senior doctors, 265 CC junior doctors, 6693 G&A senior doctors and 4306 G&A junior doctors nationally.

All resources estimated for this period are in excess, although the extent of this excess differs amongst the resources. On a per-patient-added basis, CC variables are the most limiting. The most restrictive of the CC resources is CC nurses, with the spare capacity of this only allowing for an extra 642 patients. Whereas, under the staff-to-beds ratios, the spare capacity of both CC junior doctors and CC senior doctors can accommodate an extra 2120 patients and 11,175 CC patients respectively.

### Spare capacity during the surge phase

**Fig. 2:**
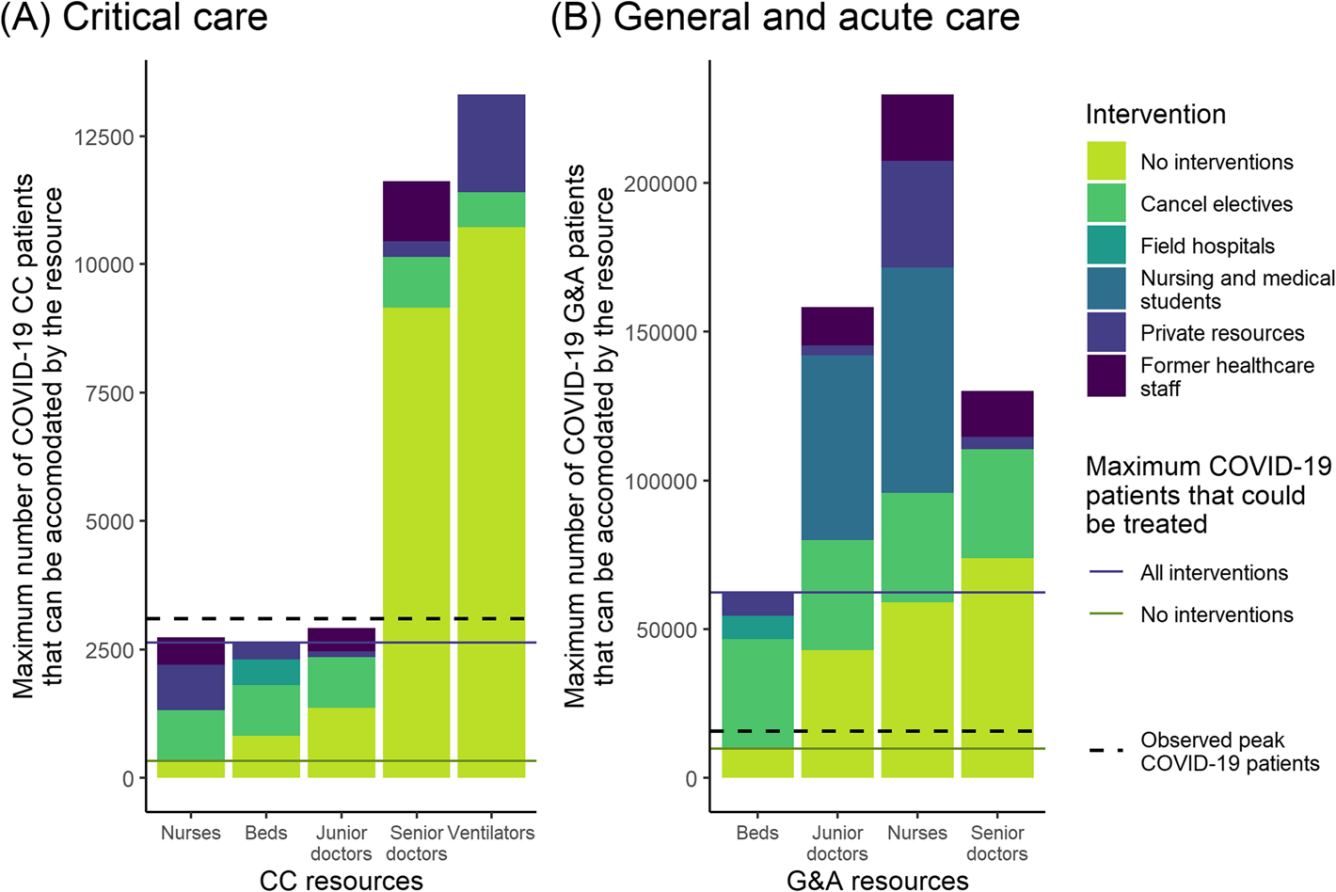
Maximum daily number of COVID-19 patients that could be accommodated by different CC (**a**) and G&A (**b**) resources with and without hospital provision interventions. *CC*, critical care; *G&A*, general and acute. Bars show the threshold of COVID-19 patients at which capacity of different resources would have been exceeded in the absence of interventions in yellow, and any additional patients under individual interventions stacked on top, so that the height of the bar represents the COVID-19 patients that can be accommodated by the combination of all interventions. Solid lines show the maximum number of COVID-19 CC (**a**) and G&A (**b**) patients that could be accommodated on any day, which is determined by the limiting resource. The dashed line highlights the observed peak number of COVID-19 patients in CC and G&A during the first pandemic wave (12th April). Note that **a** and **b** have very different vertical scales

Given estimates of baseline capacity in the absence of hospital provision interventions, and when factoring in COVID-19 related staff absence rates, up to 327 and 9769 COVID-19 patients could have been accommodated in CC and G&A care, respectively (Fig. 2). These patients would be in addition to the current patient population on any day, and we assume the recommended staff-to-beds ratios are observed. These numbers are far below the observed peak COVID-19 patient numbers of 3100 and 15,700 in CC and G&A, respectively. In CC, nurses persisted as the limiting resource at a national level, although CC beds and junior doctors would also have been insufficient to accommodate these 3100 COVID-19 CC patients. Conversely, there would have been enough daily capacity of ventilators and CC senior doctors to accommodate all COVID-19 CC patients during the surge phase even without interventions (Fig. 2a). In G&A care, only bed capacity would have been exceeded (Fig. 2b), but G&A beds had the largest deficit for the observed peak number of COVID-19 G&A patients (Table 1).

**Table 1:** Spare capacity at the pre-pandemic baseline and under alternative hospital provision intervention scenarios

Scenario	CC Beds	CC Nurses (FTE)	CC Junior Doctors (FTE)	CC Senior Doctors (FTE)	Ventilators	G&A Beds	G&A Nurses (FTE)	G&A Junior Doctors (FTE)	G&A Senior Doctors (FTE)
**No interventions**	− 2283	− 2773	− 217	403	4804	− 5931	8666	1819	3871
**All implemented interventions**	− 474 (79%)	− 359 (87%)	− 22 (90%)	568 (41%)	6430 (34%)	46,567 (885%)	42,816 (394%)	9499 (422%)	7636 (97%)
Individual hospital provision interventions									
**Cancellation of elective operations**	− 1294 (43%)	− 1784 (36%)	− 94 (57%)	469 (16%)	5230 (9%)	30,887 (621%)	16,029 (85%)	4273 (135%)	6326 (63%)
**Set-up of field hospitals**	− 1783 (22%)	−2773 (0%)	− 217 (0%)	403 (0%)	4804 (0%)	2069 (135%)	8666 (0%)	1819 (0%)	3871 (0%)
**Deployment of newly qualified and final year medicine and nursing students**	− 2283 (0%)	−2773 (0%)	− 217 (0%)	403 (0%)	4804 (0%)	− 5931 (0%)	23,805 (175%)	5981 (229%)	3871 (0%)
**Return of former healthcare staff**	− 2283 (0%)	− 2230 (20%)	− 161 (26%)	482 (20%)	4804 (0%)	− 5931 (0%)	13,099 (51%)	2660 (46%)	4909 (27%)
**Use of private hospitals**	− 1963 (14%)	− 1891 (32%)	− 203 (6%)	424 (5%)	6004 (25%)	1749 (129%)	15,879 (83%)	2041 (12%)	4144 (7%)

Note: CC: critical care; G&A: general and acute. Scenarios presented are for the observed peak number of 3,100 COVID-19 patients in CC and 15,700 COVID-19 patients in G&A. The percentage change in spare capacity of each resource for each intervention, compared to spare capacity with no interventions at peak COVID-19 patient numbers, is shown in brackets

To prevent overwhelming hospital capacity, several interventions were implemented in England across March and April 2020. The main interventions which could be quantified on a national level were those managing patient admissions and those increasing the supply of resources (Table 2). Cancellation of elective surgery and setting up of field hospitals increased available bed capacity, whereas deployment of newly qualified and final year medicine and nursing students and the return of former healthcare staff increased staff capacity. The use of private hospitals led to increases in beds, ventilators and staff.

**Table 2:** Overview of hospital provision interventions implemented in England

Intervention	Description	Effect on CC resources	Effect on G&A resources	Source
**Interventions managing admissions**				
**Cancellation of elective surgery**	Cancelling elective surgery reduces the number of beds occupied, and thereby also reduces the number of staff and ventilators required on a daily basis.	• Beds: Reduce occupancy by 30%	• Beds: Reduce occupancy by 41%	NHS Hospital Episode Statistics; Redaniel and Savovic [26, 27]
**Interventions increasing supply**				
**Set-up of field hospitals**^**a, b**^	Non-hospital sites are temporarily turned into hospitals. This increases bed numbers, but with no additional staff. In England, no details were provided about any increases in ventilator numbers solely through this intervention.	• Beds: Increase total by 500 (12%)	• Beds: Increase total by 8000 (8%)	NHS England news (03/04/20) [28], Health systems response monitor [25]
**Deployment of newly qualified/final year medicine and nursing students**^**a,b**^	Final-year medical and nursing students have their qualification process accelerated to enable them to start working immediately. They are allocated as G&A nurses and G&A junior doctors respectively.	–	• Nurses: Increase FTEs by 16,456 (51%) • Junior doctors: Increase FTEs by 4840 (47%)	BBC news (24/03/20) [29]
**Return of former healthcare staff**^**a**^	Individuals who recently worked in the health system are asked to return. This is predominantly staff who retired within the previous 3 years, but also includes individuals who left for other professions. In order to account for this fact, and also the fact that some senior staff may not wish to take on clinical decision-making responsibilities, staff are allocated across all six categories. The figures here are only for those estimated to have returned as opposed to all eligible.	• Nurses: Increase FTEs by 587 (15%) • Junior doctors: Increase FTEs by 64 (10%) • Senior doctors: Increase FTEs by 92 (10%)	• Nurses: Increase FTEs by 4822 (15%) • Junior doctors: Increase FTEs by 979 (10%) • Senior doctors: Increase FTEs by 1206 (10%)	BBC news (24/03/20) [29]
**Use of private hospitals**^**a**^	National health systems temporarily use private healthcare resources to provide public care. This increases the number of beds, ventilators and all staff categories.	• Beds: Increase total by 317 (8%) • Nurses: Increase FTEs by 955 (24%) • Junior doctors: Increase FTEs by 17 (3%) • Senior doctors: Increase FTEs by 24 (3%) • Ventilators: Increase by 1200 (15%)	• Beds: Increase total by 7683 (8%) • Nurses: Increase FTEs by 7845 (24%) • Junior doctors: Increase FTEs by 258 (3%) • Senior doctors: Increase FTEs by 317 (3%)	NHS England news (21/03/20) [30]

Note: CC: critical care; G&A: general and acute. Baseline proportions of CC and G&A were applied to data that were found to be aggregated in data sources. Staff increases account for staff sickness rates. Although further interventions involving reallocation of resources, such as conversion of operating theatres and G&A resources into CC wards and changes in staffing ratios, were also approved on a national level, these are implemented at a hospital level. As a result, their effect could not be quantified nationally and thus were not included in the analysis

^a^Full supply-side intervention package [4]

^b^Supply-side interventions deemed most sustainable in medium run [4]

Combining the interventions as parameterised in Table 2 provides an illustration of true capacity within NHS England during the surge phase. We estimate that these interventions would allow for up to 2627 and 62,267 COVID-19 patients to be accommodated in CC and G&A on any day, respectively (Fig. 2).

The most limiting resources were CC nurses, beds and junior doctors and G&A beds. The intervention that made the largest contribution to increasing their capacity was cancellation of elective surgery (Table 1, Fig. 2). Use of private hospitals and deployment of former staff were also essential to increase the capacity of CC nurses. Additionally, under the observed peak number of COVID-19 patients, setting up of field hospitals and use of private hospitals each led to large increases of around 130% in spare G&A bed capacity compared with no interventions, and deployment of medical students increased spare capacity of G&A nurses and G&A junior doctors by 175% and 229%, respectively (Table 1).

At the time of peak demand, with the combination of interventions in place, there was spare capacity in G&A beds (with a spare 46,500 beds) (Table 1) as well as capacity in staff (42,800 G&A nurses, 17,100 G&A doctors and 570 CC senior doctors) and equipment (6400 ventilators). Whilst we estimate a small deficit in CC beds, CC nurses and CC junior doctors at the time of the peak number of hospitalised COVID-19 patients, additional interventions which could not be quantified at the national level could have been used. For example, converting 474 G&A beds to CC beds and upskilling 359 G&A nurses to CC nurses would have overcome this deficit.

### Scaling up of elective surgery in the post-surge phase

As we enter the post-surge phase (Fig. 1a), attention has now turned to reintroducing elective surgery [3, 4]. We estimate that there were 989 elective patients requiring CC beds and 36,818 requiring G&A beds on an average day before the pandemic.

**Fig. 3:**
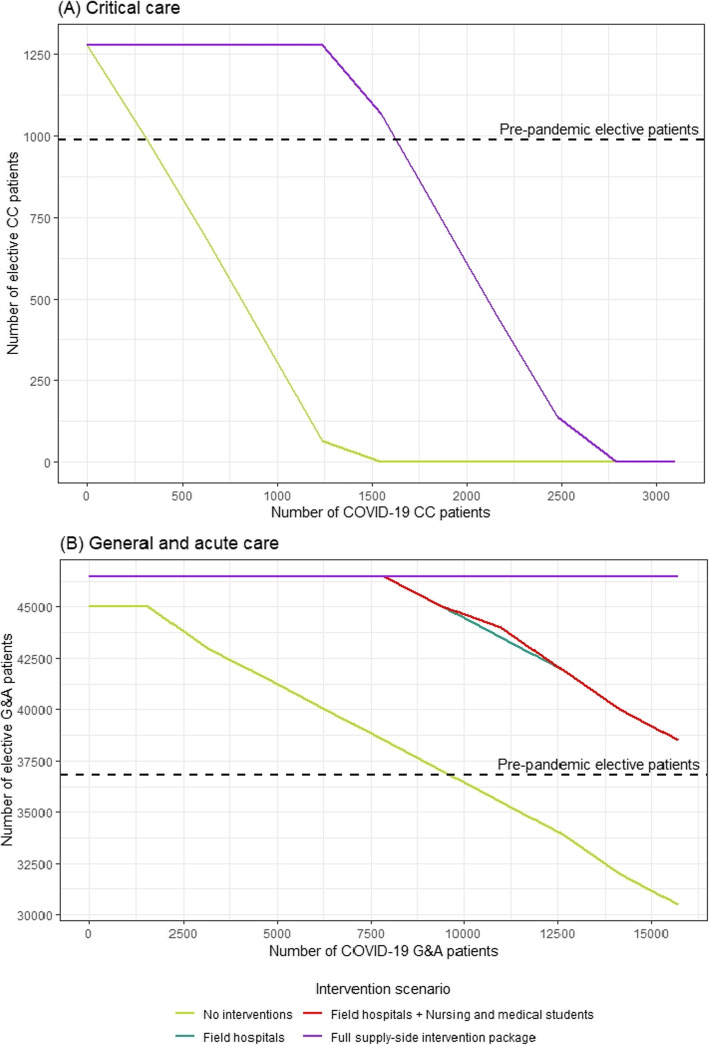
Bed availability for elective surgery considering hospital provision interventions and COVID-19 patients. *CC* critical care; *G&A*, general and acute. The relationship between the daily bed occupancy of hospitalised COVID-19 patients and beds available for hospitalised elective patients on an average day under different combinations of hospital provision interventions for **a** CC beds and **b** G&A beds. The deficit in capacity in **a** is driven by CC nurses, the capacity of which remains unchanged under all interventions except from the full supply-side package, hence field hospitals and deployment of students do not increase CC capacity above the baseline. Axis ranges cover the observed peak number of hospitalised COVID-19 patients (horizontal) and maximum average open bed numbers (vertical)

At the time of peak demand, even with the full supply-side package of interventions (Table 2), there was no capacity to treat elective patients in CC. This full supply-side package of interventions would allow 10% of elective patients requiring CC to be accommodated when COVID-19 CC patients have fallen to 2530. If no interventions were applied, then the baseline capacity would only allow accommodation of 10% of CC electives with at most 1210 COVID-19 patients in CC. To accommodate all elective patients requiring CC at average pre-pandemic levels with the full supply-side intervention package in place, the number of COVID-19 patients in CC must fall below 1550 (Fig. 3a). This is a substantial improvement upon the no-intervention scenario, in which COVID-19 patients in CC must fall below 320 for all elective patients requiring CC to be accommodated. The deficit in CC capacity is primarily being driven by nurses, which is why field hospitals, and deployment of medical and nursing students, provide no improvement over the no-intervention scenario.

However, there is greater national capacity to treat G&A patients. Without interventions, the estimated baseline capacity in NHS England could accommodate nearly 10,000 COVID-19 patients, and still treat all of the average number of elective surgery patients requiring overnight admission to G&A (Fig. 3b). The full supply-side intervention package substantially increases this capacity, allowing for demand from all G&A patients to be comfortably met even at the observed peak number of COVID-19 patients in G&A, and for more than the daily pre-pandemic number of elective patients to be accommodated (Fig. 3b). When implementing the full supply-side interventions, as above for CC, the number of COVID-19 patients that could be accommodated with all G&A elective patients rises to over 25,000.

As long as field hospitals remain operational, capacity is sufficient to meet pre-pandemic demand from all G&A patients regardless of the number of COVID-19 patients (Fig. 3b). The full supply-side intervention package could accommodate up to 46,500 elective G&A patients requiring hospital care on a daily basis, and once G&A COVID-19 patients drop to below 7500 the increase in capacity from the set-up of field hospitals is equivalent to the full supply-side intervention package. However, it is important to note that even under this intervention and with the additional deployment of students, spare capacity in G&A for COVID-19 patients was limited at the time of peak demand.

## Discussion

We developed a model to quantify hospital capacity for general and acute and critical care considering three crucial resources: staff, beds and ventilators. We used this to estimate the individual and combined impact of five interventions that were implemented in England to increase capacity to meet the demand for COVID-19 care during the surge phase: cancellation of elective surgery, setting up field hospitals, deployment of newly qualified and final year medicine and nursing students, use of private hospitals, and return of former healthcare staff. We examined potential approaches to enabling resumption of elective surgery in the post-surge phase. If no hospital provision interventions had been implemented, then capacity would have been insufficient to safely care for the peak number of 3100 hospitalised critical care COVID-19 patients which was reached on 12th April in England. The most severe constraints in critical care were numbers of CC nurses, followed by beds and junior doctors. The estimated CC capacity under the surge phase fell slightly short of the peak number of CC patients, but demand is likely to have been met using additional interventions that could not be quantified at the national level. Peak demand for G&A beds by COVID-19 patients exceeded baseline capacity, but interventions increased capacity well beyond what was eventually needed. In summary, the implementation of hospital provision interventions to manage admissions, reallocate and increase supply of resources, led to a substantial increase in capacity and has clearly contributed to ensuring access to life-supporting treatment during the pandemic surge.

Cancellation of elective surgery made the largest contribution to increasing available capacity and is an intervention that has also been implemented elsewhere in Europe [25, 31–33]. However, this may come at a substantial cost to patients whose treatments were cancelled (e.g. [34–36]). We found that elective surgery could be conducted at pre-pandemic levels if the other interventions are sustained (field hospitals, deployment of final year students, return of former healthcare staff and use of private hospitals) and there are no more than 1550 COVID-19 patients in CC beds on a given day (about 50% compared with peak demand). If this combination of interventions is not sustained, then this would only be possible for less than 320 COVID-19 patients in CC. National capacity to accommodate G&A patients is higher, with re-introduction of elective G&A patients at pre-pandemic levels being possible even without sustaining hospital provision interventions once there are less than 10,000 COVID-19 patients requiring a G&A bed. However, reducing the backlog caused by surgery cancellations requires accommodating larger numbers of elective G&A patients than pre-pandemic levels, meaning that hospital interventions are likely to need to be maintained for some time. Furthermore, it is likely that delays will have increased the complexity of treating some categories of patient, which may mean they now require CC beds rather than G&A beds.

Several tools have been developed to estimate demand for hospital care by COVID-19 patients [5–8] including the number requiring ventilation [6, 7], the different types of beds required [5, 8] or expected dates of shortfall and staff needs [5]. Our work has a different complementary objective, as it assesses how to meet demand for COVID-19 care more broadly. A strength of our study is that we evaluated the quantitative impact of interventions during March and April 2020 over baseline capacity and occupancy, by combining a review of the English response to COVID-19 surges in healthcare demand with a detailed analysis of NHS data. We then used these insights to evaluate the feasibility, in terms of capacity, of re-introducing elective surgery. Our study is one of the first to consider key human resources during the COVID-19 pandemic, including COVID-19-related staff absence. Additionally, we have made the model used in this analysis available as a user-friendly planning tool, which can assist decision makers in the adaptation of hospitals for the pandemic in different settings [24], as well as making the code publicly available on Github for others to adapt (see footnote 1).

Our analysis is conducted at the national level and thus does not consider the geographic distribution of hospital capacity, COVID-19 admissions and hospital utilisation patterns. Patterns of patient admissions may have varied spatiotemporally, with heterogeneous impact on available capacity due to variation in their average length-of-stay, but the necessary data to assess this are not currently available. Reorganisation of care within individual hospitals occurred during the surge in April, including upskilling of staff and converting operating theatres to CC wards [4], and it may be the case that recommended staff-to-bed ratios were not always able to be maintained. Furthermore, hospital infection control typically involves cohorting patients according to COVID-19 status as well as quarantining elective patients before surgery, which create local capacity challenges. As there are no consistently collected national data available on these practices, they cannot be included in the analysis. We aimed to use data from only the most robust sources, but in the absence of this, we used the best available data at our disposal.

Recent modelling predicted that temperate global regions will likely see recurrent wintertime outbreaks of COVID-19 [37], and the authors recommend increasing critical care capacity as an urgent priority. Decisions will need to be made regarding which of the interventions can be sustained and for how long, to accommodate COVID-19 and other emergency patients, address the backlog of elective patients and meet nascent demand for elective procedures. Additionally, the drop in emergency admissions may have contributed to the NHS’s ability to cope with the increase in demand [38, 39], but this may exacerbate the backlog of patients in the future.

The most severe constraint in English NHS hospitals is the number of CC nurses. This suggests that two interventions must be sustained: the deployment of former healthcare staff and the use of private healthcare provision. It will be necessary to increase the desirability of nursing to keep former healthcare staff in the profession over the course of both the pandemic and post-pandemic period. An essential intervention would be recruiting and training more CC nurses. It is possible that experienced G&A staff could be upskilled to work in CC, and their usual duties could be filled by the newly qualified and final year medical and nursing students. However, this group may require close supervision from more experienced clinical staff initially. Ongoing arrangements with private hospital providers will need to be considered. Field hospitals do not address the key constraint of CC nurse capacity but could provide overspill facilities for less severe COVID-19 patients that do not require critical nursing care, or for those requiring palliative care.

## Conclusions

The future trajectory of demand for COVID-19 care is uncertain, making it necessary to reassess the planning of elective procedures frequently; this is facilitated with our planning tool [31]. Our study demonstrates that English hospitals were successful in increasing capacity to deal with the surge in COVID-19 patients. These interventions now need to be sustained, and capacity closely monitored, to provide urgently needed care to elective patients who have waited many months for their treatments.

## Supplementary information

**Additional file 1.** Glossary. Definitions of key terms used.

**Additional file 2.** Overview table of model inputs, assumptions and how this was quantified for the analysis. Model input values, assumptions, references to data sources and how inputs were quantified for the purpose of this analysis.

**Additional file 3.** Model equations. Equations used.

## Data Availability

The data that support the findings of this study are available from multiple sources as indicated in Additional file 2, but some restrictions apply to the availability of some these data, which were used under licence for the current study, and so are not publicly available.

## Abbreviation

CC: Critical care
ESR: Electronic Staff Records
FTE: Full-time equivalents, unit that equates to employees working full time
G&A: General and acute
HES: Hospital Episode Statistics
NHS: National Health Service

## Footnotes

1 Available from: https://github.com/j-idea/england-electives
