# Supplementary material for: Adapting hospital capacity to meet changing demands during the COVID-19 pandemic
Source: BMC Med. 2020 Oct 16;18:329. doi: 10.1186/s12916-020-01781-w (PMC7565725; doi:10.1186/s12916-020-01781-w)
Supplement: Supplementary file 1 — Additional file 1. Glossary. Definitions of key terms used. [file 12916_2020_1781_MOESM1_ESM.docx]

**Adapting hospital capacity to meet changing demands during the COVID-19 pandemic**

**Additional file 1**

Ruth McCabe^1,a^, Nora Schmit^1,a^, Paula Christen^1,a^, Josh C. D’Aeth^1,a^, Alessandra Løchen^1,a^, Dheeya Rizmie^2,a^, Shevanthi Nayagam^1^, Marisa Miraldo^2^, Paul Aylin^3,4^, Alex Bottle^3^, Pablo N. Perez-Guzman^1^, Azra C. Ghani^1^, Neil M. Ferguson^1^, Peter J. White^1,5^, Katharina Hauck^1*^.

^1^ MRC Centre for Global Infectious Disease Analysis and Abdul Latif Jameel Institute for Disease and Emergency Analytics, Imperial College London, London, UK
^2^ Centre for Health Economics & Policy Innovation, Department of Economics & Public Policy, Imperial College Business School, Imperial College London, London, UK
^3^ Dr Foster Unit, Department of Primary Care and Public Health, Imperial College London, London, UK
^4^ NIHR Health Protection Research Unit in Healthcare Associated Condition and Antimicrobial Resistance, Imperial College London
^5^ Modelling and Economics Unit, National Infection Service, Public Health England, London, UK

* Corresponding author: [k.hauck@imperial.ac.uk](mailto:k.hauck@imperial.ac.uk); Imperial College St Mary’s Campus, Norfolk Place, London W2 1PG

^a^ Lead authors, guarantors

**Glossary**

| **Baseline capacity** | The baseline (average) of beds, staff and ventilators, including those occupied before COVID-19 |
| --- | --- |
| **Baseline occupancy** | The average number of beds, staff and ventilators occupied in the non-pandemic phase in the absence of COVID-19 patients. |
| **Elective patients** | Elective patients requiring hospital care on any average day pre-COVID-19 for non-urgent treatment, i.e. non-emergency, non-maternity and non-cancer. |
| **FTE** | Full-time equivalents, unit that equates to employees working full time |
| **Full supply-side intervention package** | Combination of all supply-side interventions: set up of field hospitals, deployment of newly qualified and final year medical and nursing students, return of former healthcare staff, and use of private healthcare resources |
| **G&A** | General and acute |
| **Hospital provision intervention** | A public health intervention aimed at either: managing admissions to hospitals; or re-allocating or increasing the supply of capacity in hospitals |
| **Post-surge phase** | A public health intervention aimed at either: managing admissions to hospitals; or re-allocating or increasing the supply of capacity in hospitals |
| **Pre-pandemic phase** | The period before the pandemic, i.e. before February 2020 in England |
| **Resources** | Beds, staff and ventilators |
| **Surge phase** | The period during which the pandemic peak, i.e. between March and April 2020 in England |
| **Trust** | A trust is an organisational unit, which may consist of one or more hospitals and other healthcare service facilities |
