# Supplementary material for: Adapting hospital capacity to meet changing demands during the COVID-19 pandemic
Source: BMC Med. 2020 Oct 16;18:329. doi: 10.1186/s12916-020-01781-w (PMC7565725; doi:10.1186/s12916-020-01781-w)
Supplement: Supplementary file 2 — Additional file 2. Overview table of model inputs, assumptions and how this was quantified for the analysis. Model input values, assumptions, references to data sources and how inputs were quantified for the purpose of this analysis. [file 12916_2020_1781_MOESM2_ESM.docx]

**Adapting hospital capacity to meet changing demands during the COVID-19 pandemic**

**Additional file 2**

Ruth McCabe^1,a^, Nora Schmit^1,a^, Paula Christen^1,a^, Josh C. D’Aeth^1,a^, Alessandra Løchen^1,a^, Dheeya Rizmie^2,a^, Shevanthi Nayagam^1^, Marisa Miraldo^2^, Paul Aylin^3,4^, Alex Bottle^3^, Pablo N. Perez-Guzman^1^, Azra C. Ghani^1^, Neil M. Ferguson^1^, Peter J. White^1,5^, Katharina Hauck^1*^.

^1^ MRC Centre for Global Infectious Disease Analysis and Abdul Latif Jameel Institute for Disease and Emergency Analytics, Imperial College London, London, UK
^2^ Centre for Health Economics & Policy Innovation, Department of Economics & Public Policy, Imperial College Business School, Imperial College London, London, UK
^3^ Dr Foster Unit, Department of Primary Care and Public Health, Imperial College London, London, UK
^4^ NIHR Health Protection Research Unit in Healthcare Associated Infection and Antimicrobial Resistance, Imperial College London
^5^ Modelling and Economics Unit, National Infection Service, Public Health England, London, UK

* Corresponding author: [k.hauck@imperial.ac.uk](mailto:k.hauck@imperial.ac.uk); Imperial College St Mary’s Campus, Norfolk Place, London W2 1PG

^a^ Lead authors, guarantors

**Overview table of model inputs, assumptions and how this was quantified for the analysis.**

| **Input** | **Disaggregation** | **Description** | **Symbol and value^[[1]](#footnote-2)^** | **Source** |
| --- | --- | --- | --- | --- |
| **Capacity Variables** | | | | |
| **Total number of beds** | CC;  G&A | The average daily number of beds that existed at baseline (before the pandemic). It is the sum of beds that are occupied by patients and beds that could be occupied but are not (referred to as open overnight beds in the dataset). | - *B_CC_* = 4114 - *B_G&A_* = 99569 | NHS England [9, 10] |
| **Average bed occupancy for non-COVID-19 patients^[[2]](#footnote-3)^** | CC;  G&A | The average daily number of beds that were occupied at baseline (before the pandemic). This comprises of patients recovering from elective operations, as well as emergency cases. | - $P_{CC}^{non-COVID}$ = 3297 (approx. 989 elective) - $P_{G\&A}^{non-COVID}$ = 89800 (approx. 36818 elective) | NHS England [9, 10] |
| **Observed maximum number of beds occupied by COVID-19 patients^1^** | CC;  G&A | The maximum number of COVID-19 hospitalised in English trusts on any day as of 29^th^ May 2020 (occurring on 12^th^ April 2020). Hospitalised case numbers are not split into adults and children, and so this number may slightly overestimate the true observed maximum of adult patients. | - $P_{CC}^{COVID*}$ = 3100 - $P_{G\&A}^{COVID*}$ = 15700 | NHS England [4] |
| **Number of ventilators** | Only applies to CC | The daily number of ventilators that exist at baseline (before the pandemic). It is the sum of currently used ventilators and ventilators that could be used on the day but are not. | - *V* = 8175 | The Financial Times[11], UK Government [12] |
| **Total staff (FTE)** | CC nurses (N_cc_);  CC junior doctors (JD_cc_);  CC senior doctors (SD_cc_);  G&A nurses (N_G&A_);  G&A junior doctors (JD_G&A_);  G&A senior doctors (SD_G&A_) | The average daily full-time equivalent numbers of staff at baseline (before the pandemic). | - *N_CC_* = 3939 - *JD_CC_* = 677 - *SD_CC_* = 965 - N_G&A_ = 32354 - JD_G&A_ = 10293 - SD_G&A_ = 12680 | NHS England confidential electronic staff records [13] |
| **Model Parameters** | | | | |
| **Percentage of CC patients requiring a ventilator** | COVID-19 patients;  Non-COVID-19 patients | The proportion of patients in CC expected to require a ventilator on any given day. | - ${pV}^{COVID}$= 63% - ${pV}^{non-COVID}$= 43% | ICNARC, Shahin et al [14, 15] |
| **Staffing ratios** | CC nurse (*rN_CC_*);  CC junior doctor (*rJD_CC_*);  CC senior doctor (*rSD_CC_*);  G&A nurse (*rN_G&A_*);  G&A junior doctor (*rJD_G&A_*);  G&A senior doctor (*rSD_G&A_*) | The maximum number of beds that a single staff member could safely look after. | - *rN_CC_* = 1 - *rJD_CC_* = 8 - *rSD_CC_* = 15 - *rN_G&A_* = 5 - *rJD_G&A_* = 15 - *rSD_G&A_* = 15 | Royal College of Nursing, Royal College of Physicians, Faculty of Intensive Care Medicine [16, 17, 18] |
| **Rate of COVID-19 related staff sickness or absence^[[3]](#footnote-4)^** | Nurses (*s_N_*);  Doctors (*s_D_*) | The percentage of staff absent from work for reasons related to the COVID-19 pandemic (estimates from the beginning of April). | - *s_N_* = 8% - *s_D_* = 14% | The Guardian[19], reporting on a survey conducted by the Royal College of Physicians and the Sunday Times [20] |
| **Headcount to FTE multiplier** | - | Multiplier to convert staff headcounts into staff FTEs, as official announcements of additional staff numbers are often given in headcounts. | - 0.88 | NHS Digital [21] |

1. Values are for n = No intervention scenario. [↑](#footnote-ref-2)
2. These patient numbers were both varied as part of the analysis to open elective surgery (Figure 3). [↑](#footnote-ref-3)
3. The rate of COVID-19 related staff absence was set to 0 during the pre-pandemic phase and held constant at the reported values during the pandemic. [↑](#footnote-ref-4)
