# Supplementary material for: Adapting hospital capacity to meet changing demands during the COVID-19 pandemic
Source: BMC Med. 2020 Oct 16;18:329. doi: 10.1186/s12916-020-01781-w (PMC7565725; doi:10.1186/s12916-020-01781-w)
Supplement: Supplementary file 3 — Additional file 3. Model equations. Equations used. [file 12916_2020_1781_MOESM3_ESM.docx]

**Adapting hospital capacity to meet changing demands during the COVID-19 pandemic**

**Additional file 3**

Ruth McCabe^1,a^, Nora Schmit^1,a^, Paula Christen^1,a^, Josh C. D’Aeth^1,a^, Alessandra Løchen^1,a^, Dheeya Rizmie^2,a^, Shevanthi Nayagam^1^, Marisa Miraldo^2^, Paul Aylin^3,4^, Alex Bottle^3^, Pablo N. Perez-Guzman^1^, Azra C. Ghani^1^, Neil M. Ferguson^1^, Peter J. White^1,5^, Katharina Hauck^1*^.

^1^ MRC Centre for Global Infectious Disease Analysis and Abdul Latif Jameel Institute for Disease and Emergency Analytics, Imperial College London, London, UK
^2^ Centre for Health Economics & Policy Innovation, Department of Economics & Public Policy, Imperial College Business School, Imperial College London, London, UK
^3^ Dr Foster Unit, Department of Primary Care and Public Health, Imperial College London, London, UK
^4^ NIHR Health Protection Research Unit in Healthcare Associated Condition and Antimicrobial Resistance, Imperial College London
^5^ Modelling and Economics Unit, National Infection Service, Public Health England, London, UK

* Corresponding author: [k.hauck@imperial.ac.uk](mailto:k.hauck@imperial.ac.uk); Imperial College St Mary’s Campus, Norfolk Place, London W2 1PG

^a^ Lead authors, guarantors

Model equations

The calculations underpinning the model are provided below. Calculations are analogous for general and acute (G&A) capacity and critical care (CC) capacity, except for ventilators which belong to CC only.

In accordance with the table in Additional File 2, the following notation is used:

| **Subscripts** | |
| --- | --- |
| $i$ | Refers to either General and Acute (G&A) or Critical Care (CC) |
| $n$ | Refers to intervention scenario (e.g. no intervention scenario; cancellation of electives) |
| $r$ | Refers to capacity variable (beds, nurses, junior doctors, senior doctors, ventilators) |
| **Variables** | |
| $B_{n,i}$ | Total number of beds for $i \in\{CC, G\&A\}$ and intervention $n$ |
| $P_{i}^{COVID}$ | Average bed occupancy for COVID-19 patients in $i \in\{CC, G\&A\}$ |
| $P_{i}^{COVID*}$ | The observed maximum number of COVID-19 patients occupying beds in $i \in\left\{ CC, G\&A \right\}$ as of 12th April 2020 |
| $P_{n,i}^{non-COVID}$ | Average bed occupancy for non-COVID-19 patients in $i \in\{CC, G\&A\}$ under intervention $n$ |
| $V_{n}$ | The number of ventilators under intervention $n$ |
| $N_{n,i}$ | Total nurse FTE for for $i \in\{CC, G\&A\}$ and intervention $n$ |
| ${JD}_{n,i}$ | Total junior doctor FTE for for $i \in\{CC, G\&A\}$ and intervention $n$ |
| ${SD}_{n,i}$ | Total senior doctor FTE for for $i \in\{CC, G\&A\}$ and intervention $n$ |
| ${pV}^{COVID}$ | Percentage of COVID-19 patients requiring a ventilator |
| ${pV}^{non-COVID}$ | Percentage of non-COVID-19 patients requiring a ventilator |
| ${rN}_{i}$ | Staffing ratio for a nurse in $i \in\{CC, G\&A\}$ could safely look after |
| ${rJD}_{i}$ | Staffing ratio for a junior doctor in $i \in\{CC, G\&A\}$ could safely look after |
| ${rSD}_{i}$ | Staffing ratio for a senior doctor in $i \in\{CC, G\&A\}$ could safely look after |
| $s_{N}$ | Rate of COVID-19 related nurse sickness or absence |
| $s_{D}$ | Rate of COVID-19 related doctor sickness or absence |

The primary outcome was the spare capacity of each CC and G&A resource (beds, nurses, junior doctors, senior doctors and ventilators) under different intervention scenarios, which was calculated as:

$${Spare capacity}_{n,i,r}={Resource available}_{n,i,r}-{Resource needed}_{n,i,r}$$

$$for intervention scenario n$$

$$for i \in\left\{ CC, G\&A \right\}$$

$$for r \in\left\{ beds, nurses, junior doctors, senior doctors, ventilators \right\}$$

For the different resources, this equation translates to the following:

$${Spare capacity of beds}_{n,i}=B_{n,i}-{Beds needed}_{n,i}$$

where: ${Beds needed}_{n,i}=P_{i}^{COVID}+P_{n,i}^{non-COVID}$

$${Spare capacity of nurses}_{n,i}={(\left( 1-s_{N} \right)\times N}_{n,i})-\frac{{Beds needed}_{n,i}}{{rN}_{i}}$$

$${Spare capacity of junior doctors}_{n,i}={(\left( 1-s_{D} \right)\times JD}_{n,i})-\frac{{Beds needed}_{n,i}}{{rJD}_{i}}$$

$${Spare capacity of senior doctors}_{n,i}={(\left( 1-s_{D} \right)\times SD}_{n,i})-\frac{{Beds needed}_{n,i}}{{rSD}_{i}}$$

$${Spare capacity of ventilators}_{n}=V_{n}-({pV}^{COVID}\times P_{CC}^{COVID}+ {pV}^{non-COVID}\times P_{n,CC}^{non-COVID} )$$

$$for intervention scenario n$$

$$for i \in\left\{ CC, G\&A \right\}$$

Note that the number of COVID-19 patients in CC or G&A care on a given day, $P_{i}^{COVID}$, is varied in the analysis. For the observed maximum number of COVID-19 patients to date, the spare capacity of a given resource can be calculated by setting $P_{i}^{COVID}$ = $P_{i}^{COVID*}$ (see Additional File 2 for value).

**Hospital interventions**

The baseline parameter values and sources for *B_n,i_* , *N_n,i_*, *JD_n,i_*, *SD_n,i_* and *V_n_* are summarised in Additional File 2 for *n* $\in$ *{No intervention scenario, Cancellation of elective surgery}*.

For each hospital intervention scenario increasing supply, the available capacity for each resource (*B_n,i_* , *N_n,i_*, *JD_n,i_*, *SD_n,i_* and *V_n_*) was calculated by adding the additional resources detailed in Table 2.

For *n = Cancellation of elective surgery*, the ${Beds needed}_{n,i}$ and ${Spare capacity of ventilators}_{n}$ were calculated by applying the percent reductions in occupancy to the non-COVID patients $P_{n,CC}^{non-COVID}$.

The final values for the available resources in the spare capacity equation for all modelled interventions are summarised in the following table:

|  | ***Intervention scenario n*** | | | | | | |
| --- | --- | --- | --- | --- | --- | --- | --- |
|  | **No intervention** | **Cancellation of elective surgery** | **Set up of field hospitals** | **Deployment of newly qualified/final year medicine and nursing students** | **Return of former healthcare staff** | **Use of private hospitals** | **All implemented interventions** |
| *CC resources* | | | | | | | |
| $B_{CC}$ | 4114 | 4114 | 4614 | 4114 | 4114 | 4431 | 4931 |
| $N_{CC}$ | 3939 | 3939 | 3939 | 3939 | 4526 | 4894 | 5481 |
| ${JD}_{CC}$ | 677 | 677 | 677 | 677 | 741 | 694 | 758 |
| ${SD}_{CC}$ | 965 | 965 | 965 | 965 | 1057 | 989 | 1081 |
| $V$ | 8175 | 8175 | 8175 | 8175 | 8175 | 9375 | 9375 |
| $P_{CC}^{non-COVID}$ | 3297 | 2308 | 3297 | 3297 | 3297 | 3297 | 2308 |
| *G&A resources* | | | | | | | |
| $B_{G\&A}$ | 99569 | 99569 | 107569 | 99569 | 99569 | 107252 | 115252 |
| $N_{G\&A}$ | 32354 | 32354 | 32354 | 48810 | 37176 | 40199 | 61477 |
| ${JD}_{G\&A}$ | 10293 | 10293 | 10293 | 15133 | 11272 | 10551 | 16370 |
| ${SD}_{G\&A}$ | 12680 | 12680 | 12680 | 12680 | 13886 | 12997 | 14203 |
| $P_{G\&A}^{non-COVID}$ | 89800 | 52982 | 89800 | 89800 | 89800 | 89800 | 52982 |

**Comparison of interventions**

To compare the effect of each hospital intervention on the spare capacity of a given resource, the percentage change compared to spare capacity with no interventions was calculated as follows:

$$Change in {spare capacity}_{n,i,r}\left( \% \right)=\frac{{Spare capacity}_{n,i,r}-{Spare capacity}_{no intervention, i,r}}{|{Spare capacity}_{no intervention, i,r}|}$$

$$for intervention scenario n$$

$$for i \in\left\{ CC, G\&A \right\}$$

$$for r \in\left\{ beds, nurses, junior doctors, senior doctors, ventilators \right\}$$

Further methods and assumptions

*Parameterisation of interventions*

For any intervention for which the distribution of added hospital resources across CC and G&A or senior and junior doctor strata was not reported, we applied the same distribution as derived from the data on pre-pandemic existing capacity. For example, 4% of NHS beds are CC and so 312 (4% of 8000) of private hospital resource beds were attributed to CC and the rest (7,688) to G&A.

Similarly, interventions presenting staff numbers as headcounts were multiplied by a conversion factor of 0.88 to convert to FTEs. This was determined from the ratio of headcounts to FTEs of total NHS staff in the latest available NHS workforce dataset [12].

*Analysis: The relationship between CC and G&A elective patients*

We assumed that the number of CC and G&A patients in hospital on a daily basis follows a linear relationship. Using NHS datasets, we estimated a daily average of 89,800 G&A and 3,297 CC hospitalised patients. Furthermore, using an analysis of HES data, we estimated that excluding elective patients frees-up 52,982 G&A and 2,308 CC beds. Using these two points, the relationship could be quantified. This resulted in the following equation:


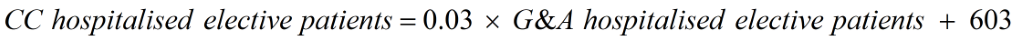


In the post-surge analysis numbers of G&A patients were varied between 52,982 and 99,482 in intervals of 500 and then numbers of CC patients were derived using this equation.
